# Human Health Risk Assessment of Toxic Elements in Farmland Topsoil with Source Identification in Jilin Province, China

**DOI:** 10.3390/ijerph15051040

**Published:** 2018-05-22

**Authors:** Fengxu Li, Jiquan Zhang, Tiehua Cao, Sijia Li, Yanan Chen, Xuanhe Liang, Xin Zhao, Junwei Chen

**Affiliations:** 1 Institute of Natural Disaster Research, Department of Environment, Northeast Normal University, Changchun 130024, China; lifx144@nenu.edu.cn (F.L.); lisj983@nenu.edu.cn (S.L.); chenyn061@nenu.edu.cn (Y.C.); chenjw585@nenu.edu.cn (J.C.); 2 Jilin Academy of Agricultural Sciences, Changchun 130017, China; liangxuanhe_2004@163.com (X.L.); zhaoxin8401@163.com (X.Z.)

**Keywords:** toxic elements, health risk assessment, farmland soils, Jilin Province

## Abstract

The presence of toxic elements in agricultural soils from anthropogenic activities is a potential threat to human health through the food chain. In this study, the concentration of toxic elements in 122 agricultural topsoil composite samples were determined in order to study the current status, identify their sources and assess the level of pollution and human health risk. The results showed that the mean concentrations of Zn, Cu, Pb, Cd, Hg and As in the farmland topsoil were 21.72, 15.09, 36.08, 0.2451, 0.0378 and 4.957 mg·kg^−1^, respectively. The spatial distribution showed that the soils were mainly contaminated by Cd, Pb and Hg in midwest Jilin but by Cu and As in the east. According to the pollution index (Pi), Nemerow integrated pollution index (PN) and Geo-Accumulation Index (*I_geo_*), Cd and Pb were the main pollutants in the soils. The occurrence of these elements was caused by anthropogenic activities and they were concentrated in the Songyuan-Changchun-Siping economic belt. There is limited non-carcinogenic and carcinogenic health risk to humans. Principal component analyses suggest the Pb, Cd and Hg soil contamination was mainly derived from anthropogenic activities in the Midwest, but all examined toxic elements in the east were mainly due to geogenic anomalies and came from atmospheric deposition.

## 1. Introduction

Soil, a non-renewable resource, is the foremost constituent of the farmland environment, hosting the agricultural production activities due to its unique characteristics [1,2], resulting from the transformation of natural regolith [3]. Farmland topsoil differs from natural soil because it is strongly shaped by agricultural practices, and has a direct influence on food quality and safety [4,5,6,7]. Furthermore, farmland soil accumulates most of the generated pollutants, especially toxic elements, due to its good absorbability [8,9]. Toxic elements in farmland soil are the subject of increasing attention and study owing to their coversion, persistence and irreversibility [10]. Large quantities of toxic elements accumulate primarily in topsoil which has the greatest ability to bond toxic elements as a result of its higher organic matter content [11].

Generally, the accumulation of toxic elements will have harmful effects, deteriorating the farmland soil and threatening ecological resources [12,13], and toxic elements can remain in farmland for decades [14]. Moreover, long-term concentration of toxic elements can also affect human health because they easily transfer into the food chain and can finally accumulate in the human body in a high concentration [15,16]. The toxic elements have different poisoning influence on the human body through their potential effects on the neurological system, kidney function, ossification process and various other organs [17]. Therefore, many studies have been devoted to soil contamination by toxic elements and management strategies for farmland soil [1,2,3,4,5,6,7,8,9,18,19,20,21,22,23,24,25,26,27,28].

There is a history of more than 100 years of heavy metal pollution study, mainly focusing on the distribution, degree of contamination, as well as the origin of toxic elements [18,19,20,21,22,23,24,25,26,27,28]. The following indexes are commonly used to evaluate soil conditions: Single factor pollution index (*P_i_*), Nemerow Pollution Index (*PN*), Geo-accumulation Index (*I_geo_*) and Potential Ecological Risk (*EI*). However, pollution indices only provide a comprehensive assessment of soil environment and ecological risk without considering human capacity. The professional health risk assessment method published by the United States Environmental Protection Agency (USEPA) has been widely used recently to assess the risk of pollutants in the environment to human health, which could help to protect public health [29,30,31,32]. The sources of toxic elements are complex and can be divided into artificial and natural sources [33]. The typical anthropogenic inputs of toxic elements in farmland soil are wastewater irrigation, atmospheric deposition and the use of fertilizers and pesticides containing toxic elements in agricultural activities [23,24,25,26,27,28].

Jilin Province plays an important role in commodity grain production in China due to its advantaged agricultural production conditions [34]. In order to assess the toxic element pollution of agricultural soils in Jilin, the main objectives of this study were to: (1) study the status of Cu, Zn, Cd, Pb, Hg and As in agricultural soils and determine the spatial distribution by a geographical information system technique; (2) analyze the factors affecting their distribution in the soils; (3) assess the degree of heavy metal pollution and soil quality by manifold pollution indices; (4) evaluate the human health risks of the toxic elements in farmland soils; (5) analyze potential sources of toxic elements in the Jilin farmland.

## 2. Materials and Methods

### 2.1. Study Area

**Figure 1:**
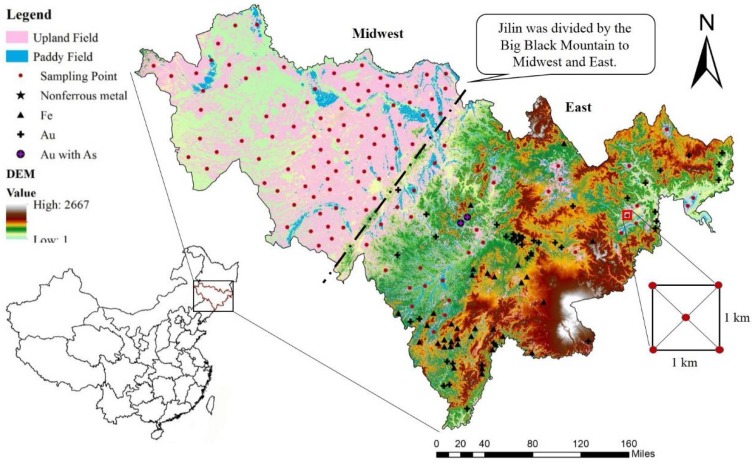
Study area and the distribution of the soil samples in the farmland.

Jilin Province (121°38′–131°19′ E, 40°52′–46°18′ N) is located in Northeast China and enjoys abundant mineral resources and is a world-famous gold maize zone. It covers a total area of 18.74 × 10^4^ km^2^, and agricultural lands account for about 40% of the total area, where 70% of the population is rural. Generally, it can be characterized in two parts: Midwest Jilin and East Jilin. There are obvious differences between the midwest and east regions, mainly due to topographic variations, especially the precipitation, which are clearly visible from the 2004 Digital Elevation Model (DEM) of Jilin Province, with 1 km resolution from the Geographical Information Monitoring Cloud Platform (http://www.dsac.cn/)There are a large number of paddy fields and dry farmland areas located in the flat midwest region of Jilin, where mainly corn and rice are grown, but only a few are scattered in the east due to the geomorphology (Figure 1). The cultivated land soil types mainly include black soil, sand and paddy soil. Jilin Province lies within a typical North Temperate Zone continental monsoon climate region, with an annual average temperate of 5 °C to 8.6 °C The annual mean precipitation ranges from 350 mm to 1000 mm and the mean evaporation in Jilin is 782 mm. The mineral resources are located mainly in the east and south of Jilin Province. At present, there is active Au and Fe mining which may be a source of toxic elements [20].

### 2.2. Sampling Collection and Analysis

Firstly, we selected the geographic coordinates of the quadrant center on the map and conducted a field sample collection campaign using a global positioning system (GPS). According to the uniform patch principle, 122 composite topsoil samples were collected from the 0–20 cm surface layer of Jilin Province farmland in 2016 (Figure 1), where each composite sample can represent the farmland soil heavy metal concentration of a circle with a 1 km radius around the collection point. Each composite soil sample consisted of five individual soil samples taken within a square plot with 1 km sides (four samples at the corners and one in the center, each of 1 kg). The individual subsamples were then completely mixed with each other by artificial turning with a spade to give a representative composite sample (1 kg) of each study point [11,35]. Due to the larger per capita cultivated area and similar cultivation methods used in different regions in Jilin Province, these 122 composite samples can better reflect the distribution of heavy metals in large scale. In the laboratory, the samples were sieved by 2 mm mesh to remove large debris, stones and pebbles after air-drying at room temperature. The pH of soil samples was determined in deionized water with a solid-liquid (S/L) ratio of 1:2.5 g·mL^−1^, using a standard combination electrode and a PHS-3C pH meter (INESA Company, Shanghai, China). According to the National Standard Method of China [36], 0.5 g of each sample soil was digested by HNO_3_-H_2_SO_4_-HClO_4_ in a 50 mL Teflon crucible with hot plate heating. The total content of Cu, Zn, Cd and Pb was determined by atomic emission spectrometry with inductively coupled plasma (ICP OES) using a iCAP 6000 Series ICP OES emission spectrometer (Thermo Fischer Scientific, Bremen, Germany). For Hg and As, another 0.5 g was digested by 10 mL HNO_3_-HCl with the HNO_3_:HCl (*v*/*v*) ratio of 1:1. The content of As determined by atomic fluorescence spectrometry (AFS), and the content of Hg determined by cold vapor atomic fluorescence spectrometry (CVAFS). The QA/QC procedures were conducted by subsequent analysis of GSS-8 certified reference material (GBW 07408-State Bureau of Metrology, Beijing, China). The recoveries ranged from 92.7% to 114.9%.

The descriptive statistics, correlation analysis (Pearson’s coefficient), and principal component analysis (PCA) were conducted using the SPSS 19.0 software (IBM, New York, NY, USA). Statistical analysis charts generated in Origin Pro 8.0 (OriginLab, Hampton, Massachusetts, USA) were used to characterize *Pi*, *PN*, *I_geo_* and *EI* of the toxic element concentrations. The spatial distributions of toxic elements were mapped by the Kriging method in ArcGIS 10.2 (Esri, RedLands, CA, USA). The data of population, population density and GDP were obtained from the Statistical Bureau of Jilin Province. 

### 2.3. Assessment Methods

#### 2.3.1. Pollution Index

The pollution index (*Pi*) and Nemerow integrated pollution index (*PN*) are useful tools for the comprehensive assessment of soil pollution degree [37]. The *Pi* represents the single pollution indices as follows (Equation (1)):

(1) $$\left\{ \begin{array}{cc} P_{i}\;=C_{i}/x_{a} & C_{i}\leq x_{a}\\ P_{i}=1+\left(C_{i}-x_{a}\right)/\left(x_{c}-x_{a}\right) & x_{a}<C_{i}\leq x_{c}\\ P_{i}\;=2+C_{i}-x_{c}/x_{p}-x_{c} & x_{c}<C_{i}\leq x_{p}\\ P_{i}\;=3+\left(C_{i}-x_{p}\right)/\left(x_{p}-x_{c}\right) & x_{p}<C_{i} \end{array}\right.$$

where *Ci* is the concentration of toxic elements in the soil, mg·kg^−1^; *X_a_*, *X_c_*, *X_p_* are the threshold concentration of the heavy metal enrichment, the low pollution level and the high pollution level, respectively, according to the Environmental Quality Standard for Soils and published by National Environmental Protection Agency of China [38].

The *PN* is used to describe the comprehensive pollution of multiple toxic elements, which could compensate for the *Pi*’s weaknesses and can be defined as follows (Equation (2)):

(2) $$PN=\sqrt{\left((Pi_{ave}){}^{2}+{\left(Pi_{max}\right)}^{2}\right)/2}$$

where $Pi_{ave}\;$ represents the average of different toxic elements’ *Pi*, $Pi_{max}\;$ denotes the maximum of *Pi* in the same sample. 

#### 2.3.2. The Geo-Accumulation Index

The geo-accumulation index (*I_geo_*) introduced by Muller [39] is used to assess the contamination levels of toxic elements resulting from anthropogenic activity. The index has been widely applied in European trace metal studies since the late 1960s, and it can be calculated by (Equation (3)):

(3) $$I_{geo}={log}_{2}\left(\frac{C_{i}}{1.5B_{n}}\right)$$

where *B_n_* is the geochemical background value in soil, referring to the China National Environmental Monitoring Centre (CNEMC) data [40]. The constant 1.5 is used as a factor to weaken the natural fluctuations in the content of a given substance in the environment [21].

#### 2.3.3. Human Health Risk Assessment

The human health risk assessment of the USEPA model and its threshold values were used to identify the exposure and assess the risk to humans. There are three main pathways of metal exposure in humans as follows: (a) inhalation of suspended particles through the mouth and nose (Equation (4)), (b) dermal absorption of trace elements in particles adhered to exposed skin (Equation (5)), and (c) direct ingestion of particles (Equation (6)). The average daily intake (*ADI*, mg·d^−1^) for non-carcinogens and carcinogens through the three exposure pathways was calculated using the following equations (Equation (7)) proposed by USEPA [41,42]:

(4) $$ADI_{inh}=\frac{CS\times IR_{air}\times EF\times ED}{PEF\times BW\times AT}$$

(5) $$ADI_{dermal}=\frac{CS\times SA\times AF\times ABS\times EF\times ED}{BW\times AT}\times 10^{-6}$$

(6) $$ADI_{ing}=\frac{CS\times IR_{soil}\times EF\times ED}{BW\times AT}\times 10^{-6}$$

(7) $$ADI_{T}=ADI_{ing}+ADI_{dermal}+ADI_{inh}$$

$ADI_{inh}$, $\;ADI_{dermal}$, $ADI_{ing}$ and $ADI_{T}$ are the average daily intake for inhalation, dermal contact, ingestion and total, respectively. *CS* is average concentration (mg·kg^−1^) of each metal, $IR_{air\;}$ (m^3^·d^−1^) and $IR_{soil}\;$ (m^3^·d^−1^) are the ingestion rate through air and soil, 7.5 and 200 for children, 15 and 100 for adult, respectively. *EF* is the exposure frequency, 350 d·a^−1^, *ED* is the exposure duration, 6a for children and 30a for adult. *PEF* is the particle emission factor, 1.36$\;\times \;10^{9}\;m^{3}$·kg^−1^. *BW*, the average body weight (kg), 15 for children and 70 for adult. *SA* is the surface area of the skin that contacts the soil (cm^2^), 2800 for children and 5000 for adult. *ABS* is the dermal absorption factor (chemical specific), 0.001%. The adsorption coefficient of the skin (*AF*) is 0.2 mg·cm^−2^ for children and 0.07 mg·cm^−2^ for adult. *AT* is the average years of exposure, 70 × 365 d [30,31,42,43]. The studied toxic elements have non-carcinogenic risk to humans but Cd and As are also identified as carcinogenic to humans. Hazard quotient (*HQ*) represent the non-carcinogenic risks of a single contaminant (Equation (8)) and it contains the risk from all three exposure pathways. Hazard index (*HI*) represents the total non-carcinogenic risks of different metals (Equation (9)) [41,44]:

(8) $$HQ_{i}=\frac{EDI_{i}}{RfD_{j}}$$

(9) $$HI=\sum^{\text{​}}HQ_{i}$$

*RfD_j_* is the oral reference doses (mg·kg^−1^·d^−1^) and it is the estimated doses of daily exposure to human that is likely not to have an appreciable risk of deleterious effects during a lifetime (Table S1). Adverse health effects should be considered cautiously, and if *HQ* or *HI* > 1, it means high risk of the toxicants with long term health hazards; if *HQ* or *HI* < 1, it indicates there is no risk. For carcinogens, the excess cancer risk (*Risk_i_*) was multiplied by the corresponding slope factor (*SF*, see Table 1) to calculate a level of excess lifetime cancer risk (Equation (10)) and it could be expressed as the total cancer risk (Risk_T_) (Equation (11)) [41]:

(10) $$Risk_{i}=EDI_{i}\times SF_{i}$$

(11) $$Risk_{T}=\sum^{\text{​}}Risk_{i}$$

Generally, if the excess cancer risks are lower than 1$\times 10^{-6}$ (a probability of 1 chance in 1,000,000 of an individual developing cancer), risks are considered negligible. If the risks are above 1$\times 10^{-4}$, they are unacceptable according to the international regulatory agencies. The value of risk between 1$\times 10^{-6}$ and 1$\times 10^{-4}$ are considered as excess cancer risks within acceptable limits.

## 3. Results and Discussion

### 3.1. Toxic Elements Concentrations and Distributions

As shown in Table 1, the soil pH ranged from 4.09 to 8.8, with a mean value of 6.49. The mean content values for Zn, Cu, Pb, Cd, Hg and As were 21.72, 15.09, 36.08, 0.2451, 0.0378 and 4.957 mg·kg^−1^, respectively. Most mean values of the metals were lower than the background values of Jilin Province soil [40] and the Natural Environmental Quality Standard values for soils in China [38], which allows us to state that toxic elements concentration in most of studied soils were at safe levels. The standard deviation values of Zn, Cu and Pb were high in relation to their mean values, caused by intense spatial variability. In particular, 13.93% of the soil samples of Cd exceeded the limit values on average 1.65 times, which indicated that part of agricultural land in Jilin were polluted by Cd. Furthermore, coefficient of variations of Zn showed a higher value (121.87%), which showed an intense spatial heterogeneity of this element. A comparison to the toxic element levels measured in other agricultural lands of China is presented in Table 2. The concentrations of Zn, Cu, Hg and As in the studied soils were lower than those from the agricultural soils which were the main areas of grain production in China, e.g., Song-nen Plain, Hebei, Hunan, Yangtze River Delta Region and Pearl River Delta. 

This could be the result of the implementation of environmental protection measures because the Zn, Cu and As mean values of Jilin were lower than their background values. However, the Cd concentration was higher than the Chinese baseline value, although it was lower than in Hunan and the Pearl River Delta. The concentration of Cd was close to the level in the Yangtze River Delta where it is mainly caused by intensive coal combustion. The average concentration of Pb was in the same range as the Yangtze River Delta and automobile exhaust emissions and industrial pollution can be indicated as the main sources of this element.

**Figure 2:**
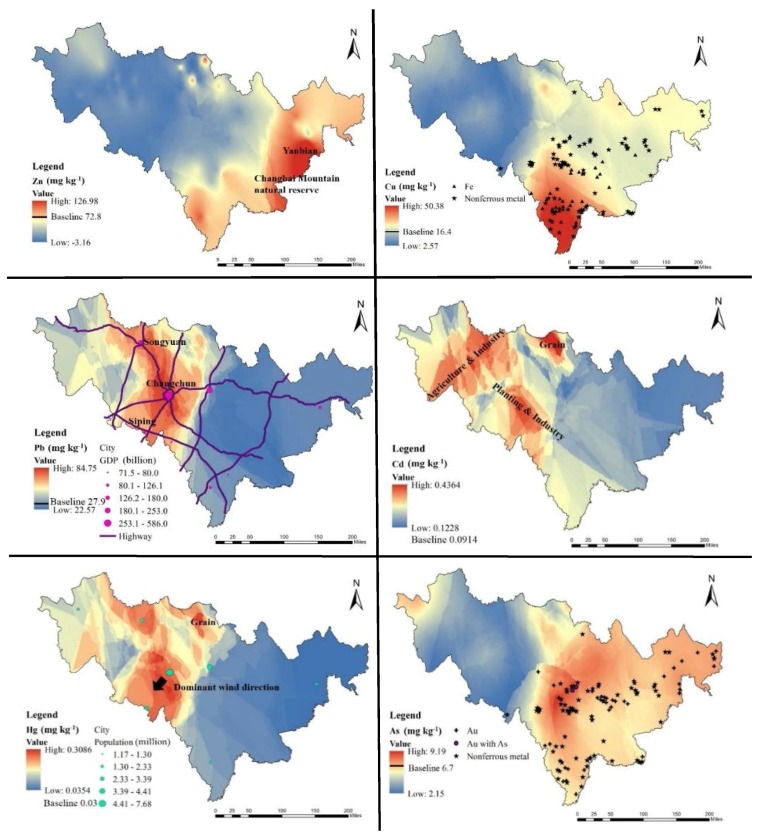
Distribution of six toxic elements in Jilin Province farmland.

The spatial distributions of heavy metal contents in the agricultural soils of Jilin Province are shown in Figure 2. The spatial distributions of Pb, Cd and Hg in soils were similar, but Cd had a relatively scattered spatial distribution. High concentrations of Pb, Cd and Hg were located in the Midwest, where are industrial corporations with developed transportation of Jilin Province and a large number of population gathered [45]. It indicated the Pb, Cd and Hg concentrations were caused by the intensive influence of anthropogenic activities with similar pollution source. Besides, the 13.93% of samples with Cd exceeding the Grade II limit (0.3 mg·kg^−1^) [38] were located in Yushu City. In this region there are many farmers are self-sufficient in vegetables, even they deliver them to the city. It should be noted the important production of vegetables in this region because Pb, Cd and Hg are the key pollutants in them. As shown in Figure 2, Zn in agricultural soils was centralized in Yanbian. The high concentration of Zn in this zone may be related to the abusing of pesticides and fertilizers, sewage irrigation, coal burning as well as originating from parent material. Cu occurred in the Tonghua City and Baishan City, in the south of Jilin Province, where are the hometown of Chinese medicine, wine, ginseng and high quality rice. The high accumulation of Cu in soil is closely related to use of Cu in pesticides, however, mining may be also another reason. The contents of As were very variable, this element occurred mainly in the East, which may be the result of geoscience or mining reasons, especially the extraction of Au ore using As compounds.

### 3.2. Assessment of Agricultural Soils Pollution

**Figure 3:**
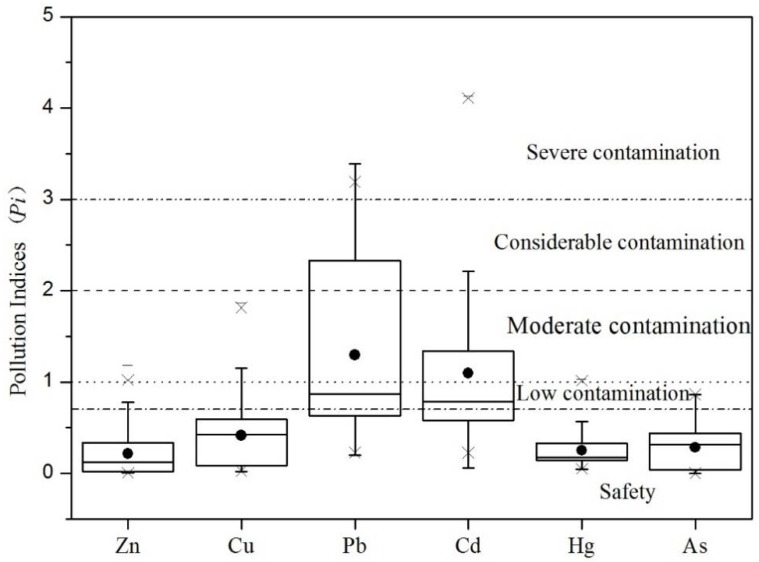
The Box-plots of pollution index for six toxic elements.

The Pi, PN and *I_geo_* have been used to assess the soil pollution status of six toxic elements. The Pi is the index used to measure that the times a single contaminant concentration exceeds national standard, according to Yang [37]. The Pi values of toxic elements were calculated (1) and presented in a box plot (Figure 3). The average Pi values of Pb and Cd were 1.30 and 1.10, which meant the studied area was moderately contaminated by Pb and Cd. These two metals, which originate from industrial activities, pesticides and chemical fertilizers, were recognized as economic development signs [4,15]. However, more than 25% of Pb samples showed a considerable level of contamination (Pi > 2), and some Pb samples were already at severe level (Pi > 3). This was most likely caused by collecting samples too close to the main transportation network and Changchun, the regional economic and industrial center city, as shown in Figure 2 (Pb). The Pi values of Zn, Cu, Hg and As were at the safety level, showing they meet the requirements of the highest Chinese soil quality standard level. In summary, the farmland soil in Jilin Province is mainly polluted by Cd and Pb.

**Figure 4:**
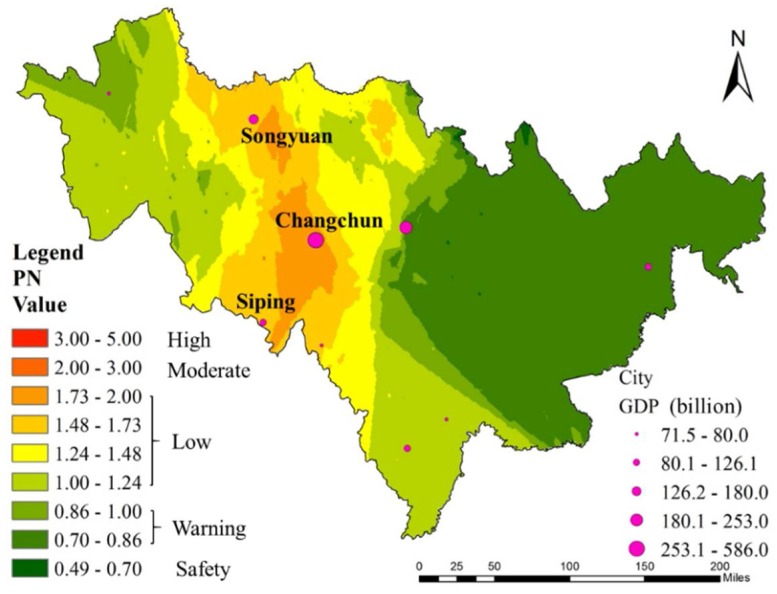
Distribution of PN in Jilin Province farmland.

The PN is a comprehensive pollution index according to the national standard (2), which indicates the environmental quality of soil. The PN of all samples ranged from 0.19 to 3.03, and the corresponding spatial distribution is presented in Figure 4. The results indicated that 8.20% of the studied area was polluted at a moderate pollution level and 34.43% at higher pollution levels, respectively. The toxic elements were mainly concentrated in the Songyuan-Changchun-Siping belt. The high value of PN in this region is inextricably linked with the development of the regional economy. The Songyuan-Changchun-Siping belt contributes to a higher GDP, as is also the transportation corridor connecting Heilongjiang and Liaoning Province. Developed industries and transportation networks often lead to toxic element pollution [24,25]. The toxic element concentration in this region exceeded the warning line. Although abundant mineral resources are located in the east of Jilin Province, there is no significant toxic element pollution due to the lack of mountain areas.

**Figure 5:**
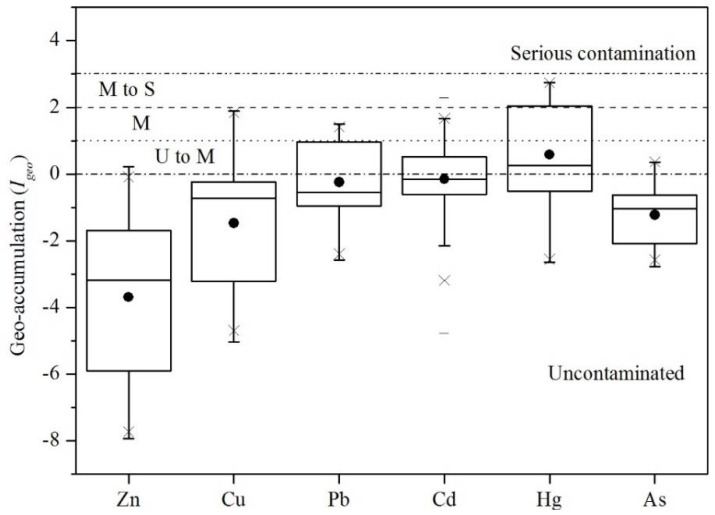
The Box-plots of geo-accumulation index (*I_geo_*), ‘U to M’—uncontaminated to moderate, ‘M’—moderate contamination, ‘M to S’—moderate to serious contamination.

The *I_geo_* was used as an index (Equation (3)) to estimate the pollution levels of toxic elements which refer to the geochemical background. The results were helpful to evaluate the inputs of toxic elements in soil from anthropogenic activities. According to Muller [39], the hierarchical results of *I_geo_* are shown in Figure 5. 

Likewise, the *I_geo_* values of Hg in 61.47% of the soil samples were greater than 0 and for 25.31% of soil samples they were larger than 1. Although Hg is at a safe level referring to the Chinese farmland soil quality standard, more than 50% of the samples had an *I_geo_* greater than 0. This means there is an external input of Hg in these areas, such as some artificial discharge of Hg [13,14]. There was 19.67% of Pb and 1.64% of Cu and 14.75% of Cd, indicating a moderate level of pollution. More than 25% of the samples were not contaminated with Pb and Cd or were moderately contaminated, signifying human activity [46]. In the case of Zn and As the samples showed no pollution, indicating that Zn and As are derived from the parent material and there is limited human input in recent years.

### 3.3. Human Health Risk Assessment

According to the USEPA equations (4–7), the exposure assessment results followed the order intake by mouth > intake of direct contact with skin > intake of breathing (Table S2). This suggests the particular toxicity of toxic elements to human health through food chain [1]. Human health assessments related to toxic elements include non-carcinogenic health risk (all studied metals) and carcinogenic health risk (just Cd and As) [44,47]. The non-carcinogenic health risk was calculated by HI (8) that is sum of non-carcinogenic hazards typically characterized by the hazard quotient (HQ). The non-carcinogenic health risk assessment results are shown in Table 3. The HI values of all examined elements were less than 1, so exposure to the toxic elements would not cause a health risk. 

Meantime, in order to evaluate the potential carcinogenic risk caused by the complex integrated effects of pollutants through ingestion, dermal adsorption and inhalation, Risk_T_ was calculated (11) (Table 4). The thresholds for carcinogenic health risk are 1$\times 10^{-6}$ and 1$\times 10^{-4}$. All values of carcinogenic health risk were below 1$\times 10^{-4}$, but there was a few As values that ranged from 1$\times 10^{-6}$ to 1$\times 10^{-4}$, suggesting that there was a carcinogenic health risk within the acceptable human range in the soil near these sampling sites.

### 3.4. Source Identification

Based on the results of distributions of toxic elements (Figure 2), our study unveiled significant spatial differences between the midwest and the east regions. The high concentration of Pb, Cd and Hg was concentrated in the midwest, but Zn, Cu and As were concentrated in the east. The topography of the midwest and east is significantly different, divided by the Big Black Mountain (Figure 1). In the Midwest are the plains, which are responsible for the major economic activities of Jilin Province with a large population density and a developed transportation net. The black belt with high productivity is mainly distributed in the Songyuan-Changchun-Siping economic belt of the midwest plains. Besides, the region with a large population consumes important quantities of coal for domestic heating. However, the east belongs to the Changbai Mountain region with the Changbai mountain volcano group near the border with North Korea. Changbai Mountain is dormant volcano. It is a major part of the Au and Fe mining activities, with sparse population and industry. Although serious toxic elements pollution of soils far from the sources of these metals is possible through atmospheric deposition, most of the important anthropogenic sources are local [33,34]. Therefore, it is indicated that the toxic element sources in the midwest and east may be different and should be analyzed separately. The values of Pearson’s correlation coefficient (*r*) for the midwest and east were thus calculated (Table 5).

In the midwest region of Jilin Province, there was a significant positive correlation between Cd and Hg (*r* = 0.99, *p* < 0.005). Similar results were found between Cu and Zn (*r* = 0.72, *p* < 0.005), and Cu and As (*r* = 0.75, *p* < 0.005). However, in the east region, there was strong correlation among Cu, Pb and Cd. Inter-element relationships in the soil matrix can provide information about heavy metal sources and pathways in the geo-environment [4,5,6,48].

**Figure 6:**
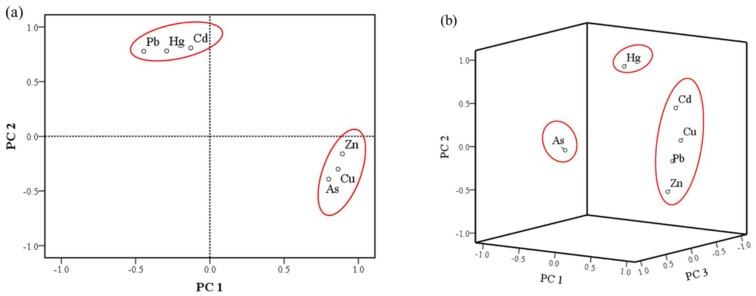
The results of PCA of six toxic elements in the agricultural soils of Jilin Province: (**a**) the Midwest; (**b**) the East.

In order to further evaluate the pollution sources of heavy metals in the study, PCA was conducted as a widespread standard procedure [19,20,21,22,23,24,25]. The PCA results are shown in Figure 6. In the midwest, based on eigenvalues (when the eigenvalue > 1.0 after varimax rotation), the PCA method resulted in a reduction of the dataset to two principal components (PCs) cumulatively explaining 76.98% of the data variance (Figure 6a). Toxic elements originating from a similar source were always grouped together with high loadings. PC1, explaining 41.36% of the total variance, was strongly and positively related to Zn, Cu and As. Most *I_geo_* values of Zn, Cu and As showed an uncontaminated level, suggesting they are mostly came from geogenic anomalies. Hence, PC1 generally represented the geogenic source of toxic elements. PC2, accounting for 35.62% of the total variance, created a related group. It demonstrated the typical contaminative elements by deposition [19,20,21]. In addition, the results of Pb, Cd and Hg *I_geo_* (Figure 5) and their high concentration (Figure 2) showed that occurrence of these elements probably resulted from anthropogenic activities. Many pesticides contain Cd, Pb and Hg [1,13,14], and Cd and Pb are also common in fertilizers [7]. Pb is a typical heavy metal in vehicle emissions, due to the use of leaded gasoline during the last century [19,28]. The presence of Hg is related to human activity and results from nonferrous metal smelting and coal burning, which represent 45% and 38% of the total release of Hg, respectively [49]. The high concentration areas of Cd, Pb and Hg were located in the Songyuan-Changchun-Siping economic belt, where the black soil belt and main traffic route are situated with a large amount of industrial production and winter heating by coal (Figure 2). The sources of Pb, Cd and Hg are likely irrigation, fertilization, pesticide application, traffic and atmospheric deposition from industrialized activities and coal burning. 

In the east region, the first three PCs cumulatively explained 87.09% of the total variance (Figure 6b). PC1, with 42.45% share, was mainly dominated by Pb, Cu, Zn and Cd, and PC2 explained 23.66% of the total variance and was related to Hg. PC3 was represented by As, accounting for 20.99% of the total variance. Pb and Cd in the east were in low concentrations, meanwhile *I_geo_* (Figure 5) of Cu and Zn showed uncontaminated area, indicating that they originated from geogenic anomalies. PC1 represented the geogenic anomaly sources of heavy metal, such as parent materials and paroxysmal eruption [5,17]. Hg and As were mostly affected by atmospheric deposition [7,18], but PCA the results divided them into two components. According to the low concentration and *I_geo_*, Hg was most likely from atmospheric precipitation from the midwest, explaining PC2. For PC3, the spatial distribution of As had a good spatial correlation with gold and iron mine distribution, but *I_geo_* of As indicated this area as uncontaminated. Therefore, the concentration of As was most likely of geogenic origin and produced by minerals, proved by the study of the deposits concentrated in the region [50,51].

## 4. Conclusions

In this study, six toxic elements were analyzed in 122 composite samples collected from farmland in Jilin Province. The mean concentrations of Zn, Cu, Pb, Cd, Hg and As in the farmland topsoils were 21.72, 15.09, 46.08, 0.2451, 0.0378 and 4.957 mg·kg^−1^, respectively. The *Pi*, *PN* and *I_geo_* index results suggested the toxic element pollution was mainly concentrated in the central economic belt of Jilin Province due to the intensive human activity. There was limited non-carcinogenic and carcinogenic health risk to humans. 

This study indicated that the toxic elements of most farmlands in Jilin were at the safety level, but there were some samples of Cd and Pb displaying relatively serious pollution in the Songyuan-Changchun-Siping economic belt, which is worth paying attention to. PCA results indicated that Pb, Cd and Hg in the midwest region stemmed from anthropogenic inputs, including irrigation, fertilization, pesticide application, traffic and atmospheric deposition from industrial activities and coal burning. The six toxic elements in the east mainly originate from geogenic anomalies. It’s worth pointing out that Hg came from atmospheric deposition and As was caused by the regional geology. The results can provide a foundation and basis to monitor and evaluate the toxic element pollution in agricultural soils by the local authorities in Jilin Province.

## Figures and Tables

**Table 1:** Descriptive statistic parameters of toxic elements in soil.

Parameter	pH	Zn	Cu	Pb	Cd	Hg	As
Mean	6.49	21.72	15.09	36.08	0.2451	0.0378	4.96
Minimum	4.09	0.45	0.75	7.00	0.0050	0.0072	1.47
Maximum	8.80	127.60	91.37	118.72	1.1150	0.1600	12.92
Standard Deviation	1.349	26.46	14.41	31.89	0.2169	0.0273	2.60
Coefficient of Variation	20.79	121.87	95.49	69.20	88.50	72.30	52.34
Jilin baseline	6.6	72.8	16.4	27.9	0.0914	0.03	6.7
Limit	-	250	100	300	0.3	0.5	40

Units of the Median, Mean, Minimum, Maximum and Limit were mg·kg^−1^, Coefficient of Variation was expressed in % and ‘-’ means no data.

**Table 2:** Average concentrations of toxic elements in soil from different regions.

Location	Site (N)	Zn	Cu	Pb	Cd	Hg	As	Reference
Jilin, China	122	21.72	15.09	36.08	0.2451	0.0378	4.96	This study
Song-nen Plain, China	20,929	55.96	18.59	22.00	0.096	0.026	8.69	Xia et al., 2014 [21]
Hebei, China	100	69.96	21.22	18.80	0.15	0.08	6.16	Yang et al., 2009 [22]
Hunan, China	6078	107.24	33.26	37.82	0.59	178.19	14.96	Liang et al., 2017 [23]
Yangtze River Delta Region, China	240	88.38	19.80	37.63	0.23	-	-	Shao et al., 2016 [24]
Pearl River Delta, China	38	84.7	33.00	40.0	0.58	-	-	Wong et al., 2002 [25]
Chinese baseline	-	74.2	22.6	26.0	0.097	0.04	9.6	CNEMC, 1990 [38]

Units of the concentrations of toxic elements were mg·kg^−1^. *N* represented the numbers of samples and ‘-’ means no data.

**Table 3:** The index of non-carcinogenic health risk.

		*HQ_inh_*	*HQ_dermal_*	*HQ_ing_*	$HI=\sum^{\text{​}}HQ_{i}$		
					Mean	Min	Max
Zn	Children	2.55355 × 10^−8^	1.29652 × 10^−5^	0.000926	0.000939	1.93 × 10^−5^	0.005514538
	Adult	1.09438 × 10^−8^	1.73641 × 10^−6^	9.92237 × 10^−5^	4.1265 × 10^−11^	8.6571 × 10^−12^	1.5737 × 10^−10^
Cu	Children	1.32892 × 10^−7^	4.49823 × 10^−5^	0.004796	0.004864647	0.000241396	0.02947863
	Adult	5.69535 × 10^−8^	6.02442 × 10^−6^	0.000514	0.000100971	2.07575 × 10^−6^	0.000592931
Pb	Children	4.59049 × 10^−6^	0.003108	0.016648	0.169589955	0.026031795	0.441769295
	Adult	1.96735 × 10^−6^	0.000416	0.001784	0.018253741	0.002801921	0.047549645
Hg	Children	4.4539 × 10^−8^	6.46113 × 10^−5^	0.005384	0.038787246	0.000318562	0.255130588
	Adult	1.90882 × 10^−8^	8.6533 × 10^−6^	0.000577	0.004196834	3.44688 × 10^−5^	0.027605484
Cd	Children	6.54999 × 10^−8^	0.001304	0.002375465	0.001541167	6.09604 × 10^−5^	0.00423861
	Adult	1.40357 × 10^−7^	4.454 × 10^−5^	0.00127257	0.000171811	3.60447 × 10^−5^	0.000655206
As	Children	4.2792 × 10^−7^	0.000105985	0.015519219	0.015625631	1.83901 × 10^−5^	0.047517758
	Adult	9.16971 × 10^−7^	7.0972 × 10^−5^	0.008314	0.008385756	9.86934 × 10^−6^	0.025501199

Units of *HQ_inh_*, *HQ_dermal_*, *HQ_ing_* and HI were mg·kg^−1^.

**Table 4:** The index of carcinogenic health risk.

		*Risk_inh_*	*Risk_dermal_*	*Risk_ing_*	$Risk_{T}=\sum^{\text{​}}Risk_{i}$		
					Mean	Min	Max
As	Children	1.92564 × 10^−10^	4.77123 × 10^−8^	7.03021 × 10^−5^	7.0333 × 10^−7^	8.2776 × 10^−9^	2.1388 × 10^−6^
	Adult	4.12637 × 10^−10^	3.19502 × 10^−8^	3.76618 × 10^−5^	3.77734 × 10^−7^	4.44562 × 10^−9^	1.1487 × 10^−6^
Cd	Children	-	-	1.49654 × 10^−6^	4.1265 × 10^−11^	8.6571 × 10^−12^	1.5737 × 10^−10^
	Adult	-	-	8.01719 × 10^−7^	8.8425 × 10^−11^	1.8551 × 10^−11^	3.3721 × 10^−10^

Units of Risk_inh_, Risk_dermal_, Risk_ing_ and Risk_T_ are mg·kg^−1^.

**Table 5:** The Pearson’s correlation coefficient (*r*) between six toxic elements (*N* = 122).

Element	Midwest (*N* = 89)						East (*N* = 33)					
	Zn	Cu	Pb	Cd	Hg	As	Zn	Cu	Pb	Cd	Hg	As
Zn	1						1					
Cu	0.724	1					0.325	1				
Pb	−0.504	−0.598	1				0.345	0.770	1			
Cd	0.076	0.028	−0.150	1			0.121	0.708	0.637	1		
Hg	0.064	0.005	−0.111	0.993	1		−0.353	0.274	0.106	0.489	1	
As	0.680	0.750	−0.625	0.025	−0.006	1	−0.428	−0.050	0.084	−0.089	0.153	1

*p* < 0.05.

## Supplementary Materials

The following are available online at http://www.mdpi.com/1660-4601/15/5/1040/s1, Table S1: RfD and SF of toxic elements for different exposure routs, Table S2: Daily average exposure of heavy metal in soil.
