# Supplementary material for: Human Health Risk Assessment of Toxic Elements in Farmland Topsoil with Source Identification in Jilin Province, China
Source: Int J Environ Res Public Health. 2018 May 22;15(5):1040. doi: 10.3390/ijerph15051040 (PMC5982079; doi:10.3390/ijerph15051040)
Supplement: Supplementary file 1 [file ijerph-15-01040-s001.pdf]

Supplementary Materials

Human Health Risk Assessment of Toxic Elements in Farmland Topsoil with Source Identification in Jilin Province, China

Fengxu Li [1], Jiquan Zhang [1,*], Tiehua Cao [2,*], Sijia Li [1], Yanan Chen [1], Xuanhe Liang [2], Xin Zhao [2] and Junwei Chen [1]

[1] Institute of Natural Disaster Research, Department of Environment, Northeast Normal University, Changchun 130024, China; lifx144@nenu.edu.cn (F.L.); lisj983@nenu.edu.cn (S.L.); chenyn061@nenu.edu.cn (Y.C.); chenjw585@nenu.edu.cn (J.C.)

[2] Jilin Academy of Agricultural Sciences, Changchun 130017, China; liangxuanhe_2004@163.com (X.L.); zhaoxin8401@163.com (X.Z.)

* Correspondence: zhangjq022@nenu.edu.cn (J.Z.); caotiehua2002@163.com (T.C.); Tel.: +86-0431-85099992 (J.Z.); +86-0431-13514405757 (T.C.)

Table S1. RfD and SF of toxic elements for different exposure routs.

Toxic Elements	RFD_{ing}	RFD_{inh}	RDF_{dermal}	SF_{ing}	SF_{inh}	SF_{dermal}
As	0.0003	0.0003	0.000123	15.1	1.5	3.66
Cd	0.0001	0.0001	0.00001	6.3	-	-
Cu	0.0402	0.04	0.012	-	-	-
Hg	0.00009	0.0003	0.000021	-	-	-
Pb	0.035	0.0035	0.000525	-	-	-
Zn	0.3	0.3	0.06	-	-	-

‘-’ means no value.

Table S2. Daily average exposure of heavy metal in soil.

Toxic Elements		ADI_{inh}		ADI_{dermal}		ADI_{ing}		ADI_T	
		**Children**	**Adult**	**Children**	**Adult**	**Children**	**Adult**	**Children**	**Adult**
Zn	Max	4.49855E-08	1.92795E-08	4.56813E-06	6.11803E-07	0.001631476	0.000174801	0.001636089	0.000175432
	Min	1.57487E-10	6.74944E-11	1.59923E-08	2.14182E-09	5.71153E-06	6.11949E-07	5.72768E-06	6.14159E-07
	Mean	7.66065E-09	3.28314E-09	7.77914E-07	1.04185E-07	0.000277826	2.97671E-05	0.000278612	2.98746E-05
Cu	Max	3.22117E-08	1.3805E-08	3.27099E-06	4.38079E-07	0.00116821	0.000125165	0.001171513	0.000125617
	Min	2.63776E-10	1.13047E-10	2.67856E-08	3.58736E-09	9.56628E-06	1.02496E-06	9.59333E-06	1.02866E-06
	Mean	5.31566E-09	2.27814E-09	5.39788E-07	7.2293E-08	0.000192781	2.06551E-05	0.000193326	2.07297E-05
Pb	Max	4.18526E-08	1.79368E-08	4.24999E-06	5.69196E-07	0.001517855	0.000162627	0.001522147	0.000163214
	Min	2.46622E-09	1.05695E-09	2.50436E-07	3.35405E-08	8.94415E-05	9.58301E-06	8.96944E-05	9.61761E-06
	Mean	1.60667E-08	6.88574E-09	1.63152E-06	2.18507E-07	0.000582686	6.24307E-05	0.000584334	6.26561E-05
Cd	Max	2.49786E-11	5.35256E-11	3.96431E-08	1.69854E-09	9.0589E-07	4.85298E-07	9.07618E-07	4.87051E-07
	Min	1.37414E-12	2.94459E-12	1.53425E-11	9.34416E-11	4.98355E-08	2.66976E-08	5.33659E-08	2.6794E-08
	Mean	6.54999E-12	1.40357E-11	1.30361E-08	4.454E-10	2.37546E-07	1.27257E-07	2.50589E-07	1.27716E-07
Hg	Max	2.0291E-09	8.69616E-10	2.06049E-07	2.75958E-08	7.35889E-05	7.88452E-06	7.37969E-05	7.91299E-06
	Min	2.53359E-12	1.08582E-12	2.57277E-10	3.44568E-11	9.18847E-08	9.84479E-09	9.21446E-08	9.88034E-09
	Mean	3.08483E-10	1.32207E-10	3.13254E-08	4.19537E-09	1.11876E-05	1.19868E-06	1.12193E-05	1.203E-06
As	Max	3.90393E-10	8.36556E-10	3.96431E-08	2.65467E-08	1.41582E-05	7.58477E-06	1.41983E-05	7.61216E-06
	Min	1.51088E-13	3.2376E-13	1.53425E-11	1.0274E-11	5.47945E-09	2.93542E-09	5.49495E-09	2.94602E-09
	Mean	1.28376E-10	2.75091E-10	1.30361E-08	8.72956E-09	4.65577E-06	2.49416E-06	4.66893E-06	2.50316E-06

Units of ADI_{inh}, ADI_{dermal}, ADI_{ing} and ADI_T are mg kg^{-1}.
